# Deep sternal wound infection and pectoralis major muscle flap reconstruction: A single-center 20-year retrospective study

**DOI:** 10.3389/fsurg.2022.870044

**Published:** 2022-07-12

**Authors:** Chen Chen, Yu Gao, Demei Zhao, Zhouji Ma, Yunyan Su, Ran Mo

**Affiliations:** ^1^ Department of Nutrition, Nanjing Drum Tower Hospital, The Affiliated Hospital of Nanjing University Medical School, Nanjing, China; ^2^ Department of Burns & Plastic Surgery, Nanjing Drum Tower Hospital, The Affiliated Hospital of Nanjing University Medical School, Nanjing, China; ^3^ Department of Cardiothoracic Surgery, Nanjing Drum Tower Hospital, The Affiliated Hospital of Nanjing University Medical School, Nanjing, China

**Keywords:** PMMF, DSWI, flap, cardiac surgery, infection

## Abstract

**Background:**

One of the most drastic complications of median sternal incision is deep sternal wound infection (DSWI), as it can lead to prolonged hospitalization, increased expected costs, re-entry into the ICU and even reoperation. Since the pectoralis major muscle flap (PMMF) technique was proposed in the 1980s, it has been widely used for sternal reconstruction after debridement. Although numerous studies on DSWI have been conducted over the years, the literature on DSWI in Chinese population remains limited. The purpose of this study was to investigate the clinical characteristics of DSWI in patients and the clinical effect of the PMMF at our institution.

**Methods:**

This study retrospectively analyzed all 14,250 consecutive patients who underwent cardiac surgery in the Department of Cardiothoracic Surgery of Drum Tower Hospital from 2001 to 2020. Ultimately, 134 patients were diagnosed with DSWI.,31 of whom had recently undergone radical debridement and transposition of the PMMF in the cardiothoracic surgery or burns and plastic surgery departments because of DSWIs, while the remaining patients had undergone conservative treatment or other methods of dressing debridement.

**Results:**

In total, 9,824 patients were enrolled in the study between 2001 and 2020, of whom 134 met the DSWI criteria and 9690 served as controls. Body mass index (OR = 1.08; *P* = 0.02; 95% CI, 1.01∼1.16) and repeat sternotomy (OR = 5.93; *P* < 0.01; 95% CI, 2.88∼12.25) were important risk factors for DSWI. Of the 134 patients with DSWI, 31 underwent the PMMF technique, and the remaining 103 served as controls. There were significant differences in coronary artery bypass grafting (CABG) (*P* < 0.01), valve replacement (*P* = 0.04) and repeat sternotomy (*P* < 0.01) between the case group and the control group. The postoperative extubation time (*P* < 0.001), ICU time (*P* < 0.001), total hospitalization time (*P* < 0.001) and postoperative hospitalization time (*P* < 0.001) in the PMMF group were significantly lower than those in the control group. The results of multivariate regression analysis showed that PMMF surgery was an important protective factor for the postoperative survival of DSWI patients (OR = 0.12; *P* = 0.04; 95% CI, 0.01∼0.90).

**Conclusions:**

Staphylococcus aureus was the most common bacteria causing DSWI, which was associated with BMI and reoperation, and can be validly treated with PMMF.

## Introduction

Since its inception in 1956, the median sternal incision has been the most commonly used incision in cardiac surgery. In patients with median sternal incisions, postoperative incision infection is one of the most important reasons for poor prognosis. Severe retrosternal infection can reach the sternum, costal cartilage, retrosternal space, bypass vessels and artificial vessels. The most dangerous complication caused by this incision is deep sternal wound infection (DSWI). The incidence of DSWI is between 0.2% and 3%. However, the mortality rate can be as high as 25.7%–52.0% (1). At the same time, DSWI also leads to prolonged hospitalization, increased expected costs, re-entry into the ICU and even reoperation (1–3). Therefore, precautionary intervention before cardiac surgery and effective treatment after the occurrence of DSWI are significant (4).

Previous studies have attempted to determine the risk factors for DSWI; previously identified risk factorsinclude obesity (5), diabetes, tracheotomy (6), use of bilateral internal mammary arteries (7) and repeat sternotomy (8, 9). After the operation,infection by *Staphylococcus aureus* (10) and other bacteria (11) results in, many clinical manifestations, including disruption of incisional wounds, massive exudation, sternum dehiscence and so on. However, there is no clear consensus on the risk factors for DSWI. Conventional treatment methods include povidone iodine irrigation (12), antibiotic irrigation (13), negative pressure wound therapy (14), debridement and other procedures, but it is difficult to completely and effectively cure DSWI. Novel evidence seems to suggest that early flap coverage after debridement has good outcomes (15). There are a variety of flap options for reconstructing sternal wounds following debridement in DSWI.

Since it was proposed in the 1980s (16), the pectoralis major muscle flap (PMMF) technique has been used increasingly clinically to treat DSWI. In recent years, we have adopted radical debridement and transposition of the PMMF to treat DSWI patients and have achieved good results.

Although studies of DSWI have been documented for many years, the literature on DSWI in the Chinese population is still limited. The present study was designed to investigate the clinical characteristics of DSWI in patients and the clinical effect of the PMMF technique at our institution. We present the following article in accordance with the STROBE reporting checklist.

## Patients and methods

### Patients

**Figure 1:**
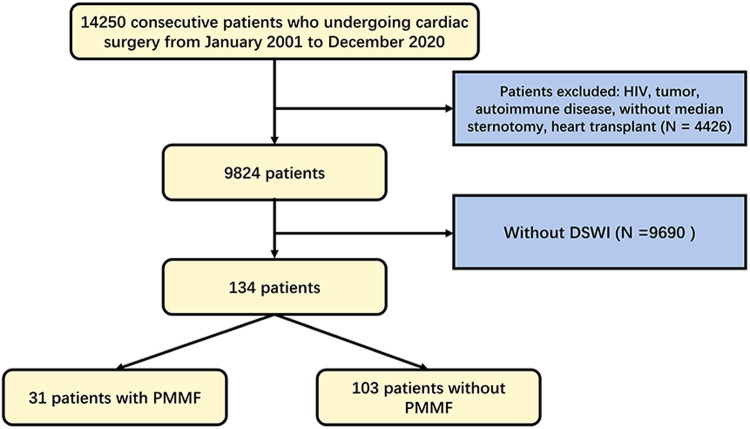
Flowchart of patient selection algorithm based on inclusion and exclusion criteria. DSWI, deep sternal wound infection. PMMF, pectoralis major muscle flap.

This study retrospectively analyzed all 14,250 consecutive patients who underwent cardiac surgery in the Department of Cardiothoracic Surgery of Drum Tower Hospital from 2001 to 2020. The research scheme was approved by the Ethics Committee of Nanjing Drum Tower Hospital (No. 2017-090-01). The study was conducted in accordance with the Declaration of Helsinki (as revised in 2013). A total of 14,250 patients were analyzed in the study (Figure 1). We excluded 4,426 patients because of HIV, tumors, autoimmune disease, the absence of median sternotomy, or heart transplantation. Ultimately, 134 patients were diagnosed with DSWI. Among these patients, 31 underwent radical debridement and transposition of the PMMF in the cardiothoracic surgery or burns and plastic surgery department because of DSWI in recent years, while the remaining patients were treated with conservative treatment or other methods of dressing debridement. The patients consisted of 74 males and 60 females, aged 23–85 years, with an average age of 57.1 ± 17.1 years. Among all patients, 32 (23.9%) underwent coronary artery bypass grafting (CABG) procedure, 45 (33.6%) underwent the valve procedure, 18 (13.4%) underwent the CABG and valve procedures, 18 (13.4%) underwent the aortic procedure, and 21 (15.7%) underwent other operations. Informed consent was obtained from the patients whose pictures were shown.

### Diagnostic criteria

DSWI is diagnosed based on a combination of clinical, laboratory and radiological findings (17). In particular, the diagnosis of mediastinitis must meet at least one of the following criteria outlined by the Centers for Disease Control and Prevention (18):

1. Positive microbial culture taken from mediastinal tissue or fluid.
2. Evidence of mediastinitis during surgery or on histopathological examination.
3. At least one of the following clinical features
  (a) Fever >38°C,
  (b) Chest pain, or
  (c) Sternal instability

**Figure 2:**
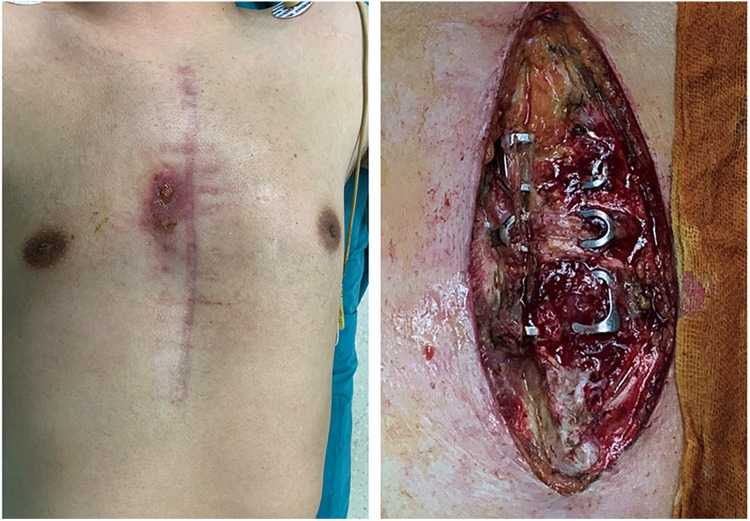
Appearance of deep sternal wound infection before operation.

Additionally, at least one of the following criteria must be met: purulent mediastinal discharge, positive microbial culture from blood or mediastinal discharge, or radiological evidence of mediastinal widening (1). We show a typical case of DSWI in Figure 2.

### Preoperative treatment

All patients had improved cardiopulmonary function and, controlled blood glucose levels (19) and received nutritional support before the operation. All incisional secretions were tested for bacterial culture, and sensitive antimicrobials were selected for anti-infection therapy according to the results of the drug sensitivity test. General principles of surgical debridement and intravenous antibiotic therapy are widely accepted for the acute phase management of DSWI. Once the diagnosis is suspected, empirical broad-spectrum intravenous antibiotics should be initiated. Once culture results are available, antibiotics should be targeted following microbiological advice (14).

### Surgical procedure

**Figure 3:**
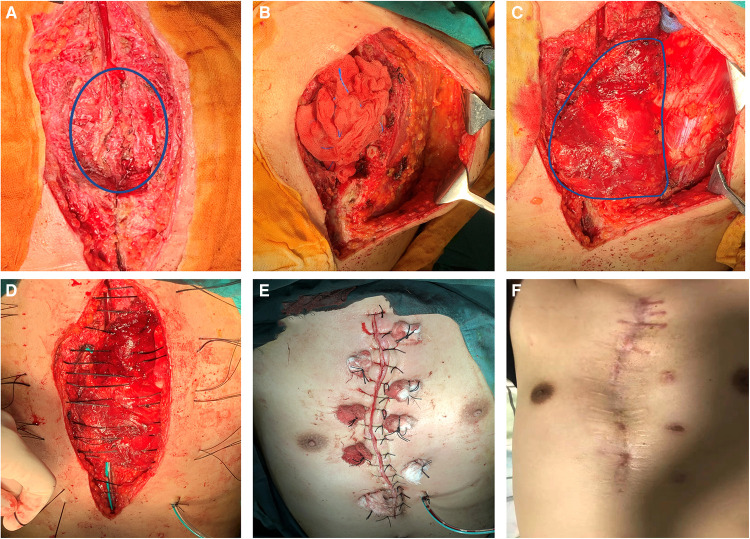
Surgical procedure and postoperative photos. (**A**) Infected sternum and implants (blue circle) were removed; (**B**) The left pectoralis major muscle was exposed; (**C**) Complete exposure of the pectoralis major muscle (blue circle) and overturning to cover the wound; (**D**) Suturing through the muscle flap; (**F**) Immediate appearance after operation; (**G**) Appearance 2 years after operation.

After general anesthesia, the patients were routinely disinfected, the incision was washed with diluted iodophor, and the infected and necrotic skin margin was removed until fresh tissue was exposed. All implants, including steel wires and memory alloy plates, were removed. The infected sternum was removed with a rongeur until normal bone was reached, and the involved costal cartilage and the bone in contact with steel wires or memory alloy plates were removed. Next, the proliferative tissue was removed. The pus was cleaned again, and the purulent exudate was scraped off; hydrogen peroxide, diluted iodophor and normal saline were used repeatedly to rinse and soak the wound, and finally the wound was rinsed with gentamicin solution (20, 21). Used gloves and instruments were replaced with clean ones. The wound was then assessed for the appropriate amount of muscle tissue necessary for closure. Based on its thoracoacromial blood supply, the entire pectoralis major muscle was mobilized following division of its insertion and rotated into the wound. Turnover flaps of the pectoralis major muscle based on perforating vessels from the internal mammary artery that did not require as much dissection were used to close the wound to just above the xiphoid process. The actual closure technique first involved elevation of the skin and subcutaneous tissues from the anterior pectoralis major fascia to just lateral to the nipple line. The pectoralis major muscle was then elevated from its lower rib origins and then divided. The muscle was elevated from lateral to medial until the perforating intercostal vessels from the internal mammary were identified. The muscle flaps were then turned into the wound and sutured to the sternal periosteum. To fill longer wounds, the pectoralis major muscles could be bisected and interdigitated If the defect area was too large, the bilateral pectoralis major could be applied, but we did not encounter such cases because the defect sizes were less than 12 cm for those in the upper 2/3 of sternum The muscle flap was sutured and fixed, and the tissue at the base of the wound after debridement was sutured together to completely obliterate dead-space. Conventional suction drains were applied (Figure 3).

In the control group,sternum suture fixation was performed after debridement under general anesthesia. After removing necrotic tissue, infected bone and foreign bodies including steel wire, titanium plate, pacing wire and hemostatic material, the wound was washed with hydrogen peroxide, diluted iodophor and normal saline repeatedly, and finally, gentamicin solution was applied to rinse the wound.

### Postoperative treatment

After the operation, patients were routinely treated with vancomycin or antibacterial therapy (22) according to the results of bacterial culture (23). The patients were generally observed in the intensive care unit for 1–2 days. After the vital signs were stable, the tracheal tubes were removed, and the patients were transferred to the general ward. The wound and drainage fluid were cultured every 2–3 days. If there was no abnormality, the drainage tube was removed, and the patients were routinely discharged.

## Statistical analysis

Categorical variables are described as frequencies (percentages) and were compared between groups using Pearson's *χ*^2^ test or Fisher's exact test. Comparisons of the continuous variables were performed by t test or nonparametric tests. Multiple logistic regression was used to identify the most significant risk factors for DSWI and the survival rate after operation. After the univariate analysis, risk factors with a *P* value < 0.1 were selected for the multivariate model. *P* values were 2-sided and a *P* value of less than 0.05 defined statistical significance. Statistical analysis was performed using SPSS 24.0 software (SPSS, Chicago, IL, USA).

## Results

Table 1 shows patient characteristics and surgical data. A total of 9,824 patients were enrolled in the study between 2001 and 2020, of whom 134 met the DSWI criteria and 9,690 served as controls. In the DSWI group, there were 74 males and 60 females, aged from 19 to 86 years, with an average age of 57.1 ± 17.1 years. In the control group, there were 5,187 males and 4,503 females, aged 21 to 90 years, with an average age of 51.4 ± 34.7 years. There were significant differences in age (*P* < 0.01), diabetes (*P* < 0.01), body mass index (BMI) (*P* < 0.01), CABG(*P* < 0.01), CABG + valve replacement (*P* = 0.01), CPB time (*P* < 0.01) and repeat sternotomy (*P* < 0.01) between the case group and the control group, while no significance was observed in sex, hypertension, isolated valve procedure, CABG + valve procedure, aortic procedure, or transfusion. Multivariate logistic regression analysis showed that age, BMI, CABG, CABG + valve surgery, CPB time, blood transfusion and repeat sternotomy were risk factors for DSWI. Indeed, BMI (OR = 1.08; *P* = 0.02; 95% CI, 1.01∼1.16) and repeat sternotomy (OR = 5.93; *P* < 0.01; 95% CI, 2.88∼12.25) were important risk factors for DSWI (Table 2). In particular, patients who underwent repeat sternotomy were five times more likely to develop DSWI than those who did not undergo repeat sternotomy.

**Table 1:** Clinical features of DSWI group and control group.

Variables	DSWI group (*N* = 134)	Control group (*N* = 9690)	*P-*value
Age(y)	57.1 ± 17.1	51.4 ± 34.7	<0.01
Gender(man)	74(55.2%)	5187(53.5%)	0.68
Diabetes	23(17.2%)	742(7.7%)	<0.01
Hypertension	39(29.1%)	2215(22.9%)	0.09
BMI (kg/m^2^)	24.9 ± 4.1	22.9 ± 4.3	<0.01
Isolated CABG procedure	32(23.9%)	1547(16.0%)	<0.01
Isolated Valve procedure	45(33.6%)	3742(38.6%)	0.23
CABG + Valve procedure	18(13.4%)	726(7.5%)	0.01
Aortic procedure	18(13.4%)	1922(19.8%)	0.06
Others	21(15.7%)	1753(18.1%)	0.47
CPB time(min)	180.1 ± 20.2	161 ± 27.3	<0.01
Transfusion	59(44.0%)	3754(38.7%)	0.21
Repeat sternotomy	17(12.7%)	271(2.8%)	<0.01

*DSWI, deep sternal wound infection; CABG, coronary artery bypass grafting; BMI, body mass index; CPB, cardiopulmonary bypass.*

**Table 2:** Multivariate conditional logistic regression results for deep sternal wound infection (DSWI).

Variables	*P-*value	Odds Ratio	95% confidence interval
Age	0.81	0.99	0.98–1.02
BMI	0.02	1.08	1.01–1.16
Isolated CABG	0.38	2.51	0.32–19.41
CABG + Valveprocedure	0.08	1.89	0.92–3.87
CPB time	0.06	1.01	1.00–1.07
Transfusion	0.24	1.35	0.82–2.20
Repeat sternotomy	<0.01	5.93	2.88–12.25

*CABG, coronary artery bypass grafting; BMI, body mass index; CPB, cardiopulmonary bypass.*

Of the 134 patients with DSWI, 31 underwent PMMF, and 103 served as controls. In the PMMF group, there were 16 males and 15 females, aged from 19 to 82 years, with an average age of 55.3 ± 16.5 years. In the non-PMMF group, there were 58 males and 45 females, aged from 20 to 86 years, with an average age of 57.5 ± 17.4 years. There were significant differences in CABG (*P* < 0.01), valve replacement (*P* = 0.04) and repeat sternotomy (*P* < 0.01) between the case group and the control group, but there was no significant difference in diabetes, hypertension, BMI, CABG and valve procedure, aortic procedure, CPB time, or blood transfusion (Table 3).

**Table 3:** Demographics and operative variables of eligible patients.

Variables	PMMF group (*N* = 31)	Non-PMMF group (*N* = 103)	*P* value
Age(y)	55.3 ± 16.5	57.5 ± 17.4	0.54
Gender(man)	16(51.6%)	58(56.3%)	0.65
Diabetes	5(16.1%)	18(15.5%)	0.86
Hypertension	7(22.6%)	32(31.1%)	0.36
BMI (kg/m^2^)	24.5 ± 4.1	25.1 ± 4.3	0.46
Isolated CABG procedure	6(19.4%)	26(25.5%)	<0.01
Isolated Valve Procedure	15(48.4%)	30(29.1%)	0.04
CABG + Valve procedure	3(9.7%)	15(14.6%)	0.48
Aortic procedure	6(19.4%)	12(11.7%)	0.27
Others	1(3.2%)	20(19.4%)	0.03
CPB time(min)	183.9 ± 21.3	177.5 ± 19.7	0.12
Transfusion	10(32.3%)	49(47.6%)	0.13
Repeat ternotomy	9(29.0%)	8(7.8%)	<0.01

*CABG, coronary artery bypass grafting; BMI, body mass index; CPB, cardiopulmonary bypass; PMMF, pectoralis major muscle flap.*

Patients with DSWI received blood cultures and exudation cultures as shown in Table 4. Of the 134 patients, 55 (41.0%) had a gram-positive bacterial culture, 42 (31.3%) had a gram-negative bacterial culture and 37 (27.6%) had a mixed infection. The most common bacteria were *Staphylococcus aureus* (24.6%) and *Acinetobacter baumannii* (18.7%). Other types of bacteria, such as *Pseudomonas aeruginosa*, *Enterobacter cloacae*, methicillin-resistant *Staphylococcus aureus* and *Enterococcus faecalis*, were also detected. The most common bacteria in the PMMF group were *Staphylococcus aureus* (29.0%) and *Acinetobacter baumannii* (25.8%). The detection rate of gram-negative bacteria (32.3%) was similar to that of gram-positive bacteria (38.7%). In addition, there was mixed infection in this group of patients (29.0%). In the control group, the most common bacteria were *Staphylococcus aureus* (23.3%) and *Acinetobacter baumannii* (16.5%). The detection rate of gram-negative bacteria (31.1%) was similar to that of gram-positive bacteria (41.7%). In addition, there was mixed infection in this group (27.2%).

**Table 4:** Pathogenic data of the DSWI patient.

Etiology	Patients with a diagnosis of etiology [*n* (%)]	
	PMMF group (*N* = 31)	Non-PMMF group (*N* = 103)
GNB	10(32.3%)	32(31.1%)
Pseudomonas aeruginosa	2(6.5%)	12(11.7%)
Acinetobacter baumannii	8(25.8%)	17(16.5%)
Enterobacter cloacae	0(0.0%)	3(2.9%)
GPC	12(38.7%)	43(41.7%)
Staphylococcus aureus	9(29.0%)	24(23.3%)
MRSA	2(6.5%)	10(9.7%)
Enterococcus faecalis	1(3.2%)	9(8.7%)
Mixed infection	9(29.0%)	28(27.2%)

*GNB, gram negative bacteria; GPC, gram positive bacteria; MRSA, Methicillin resistant Staphylococcus aureus.*

**Figure 4:**
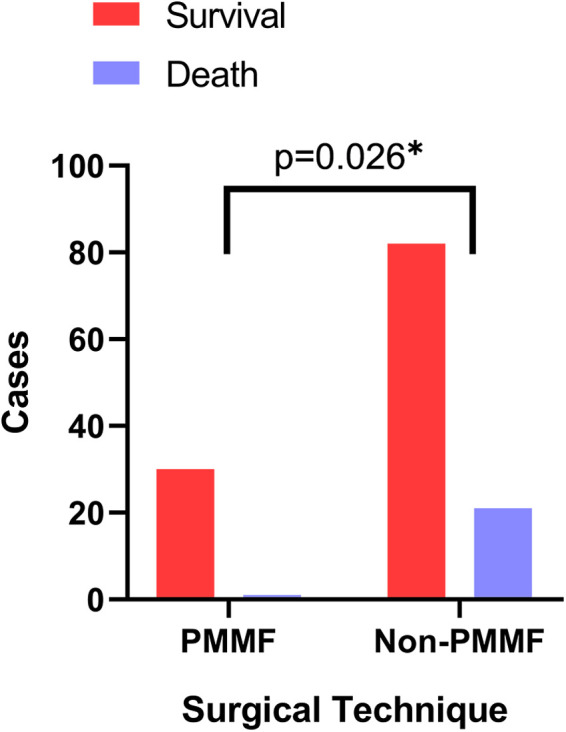
Comparison of survival rates between the PMMF group and the control group. PMMF, pectoralis major muscle flap. *: *P* < 0.05.

The postoperative results were as follows (Table 5). The postoperative extubation time (*P* < 0.001), ICU time (*P* < 0.001), total hospitalization time (*P* < 0.001) and postoperative hospitalization time (*P* < 0.001) in the PMMF group were significantly lower than those in the control group, while no significant difference was detected in 24-hour drainage (Table 5). In addition, the survival rate of the patients in the PMMF group (96.8%) was significantly higher than that of the patients in the control group (79.6%) (*P* = 0.026) (Figure 4). In the PMMF group, 28 and three cases experienced primary healing and delayed healing of the skin incision, respectively. None of the patients experienced subcutaneous hematoma or bleeding that required reopening and drainage. All patients recovered and were discharged. After discharge, none of the patients reported abnormal movement of the upper limbs or obvious abnormalities in the appearance of the chest. The patients were followed up for an average of 6 months following flap surgery. None of the patients experienced DSWI recurrence. No postoperative complications, including discomfort, abnormal movement of the upper limbs, or chest deformity, were reported by all any of the 31 patients.

**Table 5:** Results of operation in two groups.

Characteristics	PMMF group (*N* = 31)	Non-PMMF group (*N* = 103)	*P-*value
Time to extubation, h	6.7 ± 3.8	20.4 ± 26.8	<0.001
ICU time, d	1.7 ± 1.7	7.5 ± 8.5	<0.001
24-hour drainage,ml	450.0 ± 528.5	708.9 ± 674.8	0.210
Hospitalization time, d	29.9 ± 29.9	49.2 ± 34.2	<0.001
Postoperative hospitalization time, d	21.4 ± 17.1	41.9 ± 28.6	<0.001
Survival rate	30 (96.8%)	82(79.6%)	0.026

*ICU, intensive care unit; PMMF, pectoralis major muscle flap; h, hours; d, days.*

The results of multivariate regression analysis were as follows (Table 6): PMMF surgery was found to be an important protective factor for the postoperative survival of patients with DSWI (OR = 0.12; *P* = 0.04; 95% CI, 0.01∼0.90). The risk of death in patients undergoing the PMMF procedure was only 12% of that in the control group. Other factors included age, BMI, type of operation and CPB time before the operation, which were not statistically significant in the multiple regression analysis.

**Table 6:** Multivariate conditional logistic regression results for survival rate after operation.

Variables	*P*-value	Odds Ratio	95% confidence interval
Age	0.91	0.99	0.97–1.03
BMI	0.86	1.01	0.90–1.13
PMMF	0.04	0.12	0.01–0.90
Isolated CABG	0.33	2.51	0.32–19.41
Isolated valve procedure	0.41	1.78	0.46–6.93
CABG + Valve procedure	0.16	4.88	0.54–44.37
Aortic procedure	0.40	0.54	0.13–2.28
Others	0.97	1.02	0.21–5.08
CPB time	0.68	0.99	0.97–1.02

*CABG, coronary artery bypass grafting; BMI, body mass index; CPB, cardiopulmonary bypass; PMMF, pectoralis major muscle flap.*

## Discussion

DSWI is the most serious postoperative complication caused by a median sternal incision. Robinson et al reported that the incidence of DSWI was 1.3% in 12,000 patients who underwent cardiac surgery from July 2001 to June 2005 (24). We found that the incidence of DSWI is 1.36% at our institution and is comparable with the report above. Once this condition occurs, it will quickly infect the retrosternal mediastinum. Although for some very early patients, there is a chance to heal after removing sutures and exposing the wound by debridement, most wounds were not able to heal after repeated debridement and repeated infection, which eventually lead to complete wound dehiscence. Expansion and contraction by breathing further increases the difficulty of wound healing, eventually leading to deeper infection. Once bacteria are implanted in the heart or on implanted materials such as sutures, artificial blood vessels, and artificial valves, they will quickly lead to the deterioration of cardiac function and even death (25).

The occurrence of DSWI is related to many factors. Floros et al. determined that the risk factors for DSWI consisted of smoking, re-exploration, oral hypoglycemic drugs, emergency surgery, obesity, IABP and transfusion (9). In addition, Pilarczyk et al. identified that percutaneous dilatational tracheotomy within 48 h after cardiac surgery was associated with mediastinitis (26). In our study, we found that high BMI and secondary surgery were the most important risk factors. Although it was not identified as a significant risk factor, age was a significant variable between the DSWI group and the control group, as the DSWI group was older than the control group. Patients who have higher BMI are in danger of sternal instability. Therefore, sternal instability may contribute to the development of DSWI in elderly patients who receive cardiac surgery *via* a median sternotomy. Furthermore, our results showed that reoperation was a risk factor for DSWI after surgery. Patients who undergo reoperation often suffer from a worse tissue situation and need more time, usually because of bleeding or unstable hemodynamics. These situations probably significantly increase DSWI risk, but an additional randomized control trial is needed to verify this conjecture (9, 27–29).

PMMF was first described for use in the setting of DSWI by Jurkiewics in the 1980s (16). It can be used as either a unilateral or bilateral flap depending on the defect size. Double-breasting flaps can improve sternal stability and further obliterate dead-space. In contrast, simple debridement without flap coverage may contribute to chest instability. PMMF is a reliable flap for superior 2/3 sternal defects. It can be used in the absence of the IMA as an advancement flap, or as a perforator flap where the IMA is present to increase its excursion for the reconstruction of larger defects (14). Meanwhile, rectus abdominis (RA) muscle flaps are ideal for use in reconstructing the lower third of the sternum.

The most common bacteria infecting the wounds were *Staphylococcus aureus* (24.6%) and others, such as *Acinetobacter baumannii* (18.7%), and many patients had mixed infections (27.6%). There are many different methods for the treatment of DSWI. In addition to routine anti-infection and debridement, continuous negative pressure wound therapy is a more common method; however, based on our experience, the effect is good for superficial soft tissue infection, but for deep soft tissue infection, the failure rate is reported to be approximately 12.5%–50.0%, which makes it difficult to achieve satisfactory clinical effects. We have found that for patients with superficial tissue infection, continuous negative pressure wound therapy can be used at the same time after thorough deep debridement, which can yield a better clinical effect.

Local gentamicin was introduced in cardiac surgery several years ago and has proven effective in the prevention of DSWI. A review of literature on the application of local gentamicin in the prevention of DSWI revealed 4 randomized trials, of which 3 showed a beneficial effect (30). Topical gentamicin was also used. However, a recent randomized controlled trial reported that addition of local gentamicin in the treatment of DSWI did not result in a shorter length of stay (31). This was inconsistent with previous findings. Nonetheless, only 41 patients were included in the study. Larger randomized controlled trials need to be conducted.

Regarding the time of debridement and flap surgery, the previous DSWI guidelines suggested that the earlier the intervention, the better (29, 32–34). We have practiced this view in the clinic. After the discovery or confirmation of DSWI in any patient, the operation should be carried out as soon as possible on the premise that the cardiopulmonary function is normal and the patient's general condition can tolerate the readministration of general anesthesia (35). During the operation, all implants outside the heart and aorta should be excised as much as possible, and all suspected infected tissues should be removed thoroughly without hesitation to ensure radical treatment.

Some studies believe that the use of negative pressure wound therapy (NPWT) after PMMF can effectively reduce the probability of infection. In our clinical practice, we also tried to use NPWT and achieved good results, but because of the small number of cases, this was not analyzed separately. NPWT can keep the wound dry continuously, reduce the chance of bacterial growth, and reduce the burden on medical staff without the necessity of changing dressings every day. Effective drainage by NPWT can also promote the rapid healing of wounds. Although it may increase the cost of hospitalization, considering the reduced infection risk and hospitalization time, we believe that NPWT should be recommended as a routine postoperative and even preoperative treatment.

The vast majority of people, even those of who are engaged in manual work, do not have well-developed pectoralis major muscles, unless they are deliberately exercising it. In fact, although Chinese people have come to gradually realize the importance of fitness, it has not been practiced for a long time (36, 37). Therefore, according to our follow-up, there was no significant effect on upper limb movement. In many cases, the sternum was removed completely that we could see the beating of the heart across the chest wall. The latest consensus is that when bone quality post-debridement is adequate, sternal fixation with rewiring or plating may result in greater sternal stability than flap closure alone, and should be considered as an option, although flap closure remains the gold standard option (14). The stability of the ribs mainly depends on the spine. When patients focused on protection, for example, within 8 weeks after the operation, he or she was asked to avoid excessive weight-bearing of the upper limbs and twisting or rotating the upper body, none of which was shown to affect the patient's daily life.

Although the findings were statistically supported with a large sample size, there were some limitations that may have affect the findings. First, this is a retrospective study, which cannot account for the effect of unknown variables. Second, our research time span was very long, the operation was not performed by the same doctor, and the choice of operation had a lot to do with the reconstruction techniques, personal experience and patients' wishes at that time. Thus, a prospective randomized controlled trial should be conducted to analyze the characteristics of DSWI in the Chinese population.

## Conclusion

Staphylococcus aureus was the most common bacteria causing DSWI, which was associated with BMI and reoperation, and the PMMF technique was shown to be a valid treatment in our study. Among 31 patients, 90.3% healed primarily and no obvious complications were reported.

## Data Availability

The raw data supporting the conclusions of this article will be made available by the authors, without undue reservation.

## Reporting checklist

The authors have completed the STROBE reporting checklist.
